# Cancer and Anorexia Nervosa in the Adolescence: A Family-Based Systemic Intervention

**DOI:** 10.1155/2011/769869

**Published:** 2011-08-07

**Authors:** Gabriella De Benedetta, Ida Bolognini, Silvia D'Ovidio, Antonello Pinto

**Affiliations:** ^1^Hematology-Oncology and Stem Cell Transplantation Unit, Department of Hematology, “G. Pascale” Foundation, IRCCS, 80131 Naples, Italy; ^2^Pediatric Oncology Service, Pediatric Department, Second University of Naples (SUN), 80138 Naples, Italy

## Abstract

*Objective*. Anorexia nervosa is difficult to diagnose in cancer patients since weight loss, aversion for food, and eating disturbances are frequent in patients undergoing chemotherapy and radiotherapy. Nevertheless, efforts are mandatory to recognize and manage this condition which may occur also in cancer patients with a special regard to adolescents. *Methods*. Through the clinical history of Anna, a 15-year-old adolescent with advanced cancer, we describe the effectiveness of a family-based systemic intervention to manage anorexia nervosa occurring in concomitance to osteosarcoma. *Results*. Through a two-year psychotherapy period involving different techniques applied to the whole family such as family genogram, family collage, and sculpture of family time, Anna was relieved from her condition. *Conclusions*. Upon early diagnosis and appropriate treatment, anorexia nervosa can be effectively approached in adolescent cancer patients. The presence of a life-threatening medical condition such as cancer may provide motivation for a patient to control disordered eating behavior in the context of an appropriate family-based systemic intervention. The general frame of anorexia occurring in cancer-bearing adolescents is reviewed and discussed.

## 1. Introduction

Anorexia nervosa is a well-defined disorder characterized by weight loss, loss of the desire to eat, fear of gaining weight, distorted body image, and denial of physical consequences deriving from a sharply reduced food intake [1–4]. Disturbances of eating behavior are also common in cancer patients throughout different phases of their disease. Approximately 50% of tumor-bearing patients may display abnormalities of eating behavior at the time of first diagnosis while the prevalence of feeding disturbances in terminally-ill patients is estimated between 65% and 74% [5, 6].

In cancer patients disturbances of eating behavior may have a multifaceted etiology spanning from transient anorexia secondary to the psychological distress of cancer diagnosis to anorexic phases associated to treatment side effects (mucositis, alteration of taste, nausea, and vomiting) which may ultimately lead to the cancer anorexia-cachexia syndrome (CACS) of advanced tumors [7, 8]. This latter has been suggested to represent a distinct entity with unique clinical characteristics associated to abnormalities in circulating inflammatory proteins and cytokines [7, 8]. In general, several physical factors related to disease, toxicity of administered drugs, and cancer-associated pain may lead patients to refuse foods [5–7]. Seminal studies from Holland et al., have established a careful distinction between phases in which anorexia may manifest in cancer patients: at the beginning of the disease, a stage characterized by a non-acceptance of the diagnosis and of the associated physical changes and by an alteration of the whole lifestyle due to treatments and/or hospitalization, concomitantly or after chemoradiotherapy or following digestive tract surgery, during the end stages of terminal disease progression [9]. So, except at diagnosis, when a psychological component can be present, anorexia in cancer patients is mostly regarded as a direct result of physical insults linked to both disease and treatments [9, 10]. These situations, however, are well outside the psychopathological area whose borders are crossed only when the refusal of food becomes a permanent behavior supported by specific beliefs [1–4]. 

As a matter of fact, anorexia nervosa is difficult to diagnose in patients undergoing cancer treatments. Weight loss, the refusal of food, or its reduced intake may be linked to treatment side effects (nausea, vomiting, mucositis, and alteration of taste) and/or represent an emotional response of patients towards a situation they refuse to accept [8–11]. Many children with cancer, for example, may refuse food during the hospitalization as a sign of non-acceptance of their condition, but rapidly resume their physiologic eating behavior once back home. As a further example, during chemotherapy it is not possible to identify the absence of menstruation, one of the main diagnostic criteria of anorexia nervosa, since many young patients receive ovariostatic therapy, usually through the use of birth control pills [1–4]. So the boundary between overt psychopathology and psychoemotional distress can be very subtle in the setting of neoplastic disorders.

Unfortunately, the overlaps between cancer-related anorexia and anorexia nervosa have been poorly explored and the issue is underrepresented in the scientific literature. While cases fulfilling all the diagnostic criteria for anorexia nervosa have been usually described in long-term cancer survivors, the occurrence of this disorder concomitantly with cancer diagnosis or during the disease course has been rarely reported [12–14]. These cases, obviously, also need to be separated from those in which the presence of a brain tumor close to the limbic system or other areas involved in appetite regulation could be causative for eating disorders [15] and from situations in which tumor-related symptoms are mistakenly diagnosed as an eating disorder [16].

We report here the case of an adolescent in which anorexia nervosa has occurred during the postsurgical stage of her cancer treatment. Results are discussed in the context of the family-based systemic intervention adopted to manage the situation and within the general frame of anorexia occurring in cancer-bearing adolescents. The patient is given the name Anna in this report to protect her identity.

## 2. The Story of Anna

Anna was 15 years old when she first knew about her osteosarcoma. She was a nice, only child, very clever and successful at school, with a preferential attitude towards cognitive rather than emotional abilities. She was paying careful attention to her body image, such as all young adolescent girls, but without any anxiety; she was used to limit over assumption of hyper caloric foods but without specific dietary restrictions. After diagnosis she was told that her treatment was going to consist of courses of chemotherapy to be delivered before and after major surgery to the lower limb with implant of right femur endoprosthesis. Being, in a postmenarcheal phase, she was given ovariostatic treatment before chemotherapy. As usual practice at our institution, Anna and her family were provided psychological support at diagnosis and throughout the entire disease course as inpatient and during outpatient chemotherapy sessions. Rationalization was the psychological defense mechanism the patient most used against her disease. During presurgical chemotherapy Anna appeared very compliant; she never complained about illness or hospitalization and rather tried to make other patients happy, whenever they were sad, and continuously supported them to react against cancer. In the ward she played the same role she acted in her family: a responsible, happy, and cheerful girl, always ready to give support to others and unwilling to recognize her needs and difficulties. She was always accompanied by an aunt (her mother's sister) during hospital admissions and outpatient therapy, since, in Anna's wording “…she was able to give her more peace than her mother.” As emerged from later psychotherapy sessions she was trying to protect her mother but, at the same time, she did not feel protected by her. 

At the beginning, Anna's refusal of food was irregular and apparently related to administration of chemotherapy. Starting from the early post-surgical phase, however, she displayed a progressive reduction in food intake which rapidly turned to a severe food-restricting behavior. Since in Mediterranean culture “good eating” is associated to the idea of “staying well,” parents and relatives always pay attention to feeding habits of their sick loved ones with the idea “good eating” will help to heal. However, parents and relatives of neoplastic children/adolescents are educated by the hospital staff not to force patients to eat too much and to respect them when they refuse food because they do not feel well. Furthermore, during the treatment, which often involves corticosteroids, the appetite may become voracious and so parents are asked to control young patients' diet. They could then get accustomed to dietary patterns which are completely different from usual and often characterized by alternate periods of uncontrolled appetite and starvation. For such reasons the severity of Anna's refusal of foods at home was initially underestimated by family members and not reported to her physician. In addition, weight loss occurred gradually, was “hidden” through appropriate clothing, and associated to secretive eating behaviors. During courses of post-surgical chemotherapy, Anna started to appear unusually concerned about her body image, with a special focus on her upper limbs and abdomen “…I'm developing fat arms…,… do you think I'm getting an enlarged belly…”.

It was only following a specific enquiry from attending doctors and after that Anna's chemotherapy was postponed due malnutrition that parents and other family members realized the severity of her situation. Anna and her family agreed, however, to start a family-based outpatient psychotherapy program, the patient received dietary supplements to allow a rapid weight gain and resumed chemotherapy with a three-week delay. While on psychotherapy Anna completed her chemotherapy. At subsequent outpatient controls, however, she still had amenorrhea and a progressive weight loss was again recorded up to <85% of expected weight. Admission to an “eating disorder unit” was discussed during a psychotherapy session but both Anna and her therapist agreed on avoiding it given the long hospitalization periods already experienced by the patient for treating her cancer and rather continuing with sessions. Psychotherapy lasted two years and at last followup Anna, 8 months from end of psychotherapy, gradually recovered her body weight showed no tumor recurrences, and regained her menstrual cycle. At the writing of this report she is attending the first year of medical school willing to become a pediatric oncologist.

## 3. Results

While a continuous psychological support was offered to Anna since diagnosis, the intervention was scheduled according to the therapeutic program and provided during hospital admissions and outpatient sessions. In contrast, development of anorexia prompted the activation of an outpatient psychotherapy program with specific times and spaces. Parents and relatives, anguished over malnutrition-related delays of chemotherapy, declared themselves available for a family psychotherapy involving meetings with both the whole family and its subsystems (parental couple and the patient). The psychological work was based on different approaches including definition of a three-generation family genogram, family collage, and sculpture of family time [17–21]. Analysis of genogram depicted a family organization in which Anna's mother occupied a very marginal role being also very poorly committed to attend her daughter, which substantially grew up with grandparents. It was very often Anna's aunt (her mother's sister) to accompany the girl to individual meetings or to telephone if they had to schedule or cancel an appointment. There was a great confusion of roles in the management of Anna, where it was not clear who was the parent. Anna's grandmother looked after her cooking for her and keeping her things in order, while her parents paid only attention to the economic situation, her aunt took care of the aspects of the disease and the uncle accompanied her to go out with friends. The whole family appeared compact around her but it seemed that nobody was ready to assume a full responsibility for this teenager. The therapist temporary played this role which was then gradually returned to the parents once they felt ready and when Anna showed the desire and the need to share her life with parents, feeling them as their real parents.

At the beginning, the desperate need for help of Anna's parents did not correspond to a real willingness to change. This was mirrored by their apparent difficulty to attend the meetings mainly justified by logistic issues. This is understandable by also considering that both anorexia and cancer are associated to a gloomy prognosis which rendered the anguish of death intolerable in the family system. It was also the need to protect themselves from excessive suffering that lead Anna's parents to initially disregard the severity of anorexic symptoms. 

The concept of transformation in family psychotherapy is linked with wide-ranging movements that are realized with time. The therapist needs to draw a specific road map and know how to facilitate the small movements that bring the family to achieve the planned goals, helping its members not to feel threatened by all these big changes [22, 23]. To overcome the impasse that anguished Anna's parents, preventing them to benefit from the help offered, we have used the patient herself as the main instrument for our support. Anna's absolute power over her family added to her motivation to begin psychotherapy has been crucial factors in getting the participation of her parents. The alliance with the patient, essential to engage the whole family, has been possible also because the psychological support occurred in the context a significant relationship previously built. Although some people with anorexia can be aware of their “slimness,” most typically deny the serious consequences of malnourishment on their physical health. Only the serious interference of malnutrition with treatments added to her willingness to fight cancer have induced Anna to become aware of the food problem. So, at this point, it has been possible to support her desire to fight cancer and not to die. The collaboration between doctors and psychotherapists has been particularly fruitful in releasing a complex situation.

Psychotherapy with Anna has been rich and varied. We have faced a situation in which the trauma of cancer, as we have seen, becomes part of a complex family frame characterized by poor differentiation, delegation of care, loyalties, confusion of roles, and an extreme denial of the change. The alteration of the plans and the generational confusion of roles were reflected by the organization of the family space: Anna slept in her grandparents' home, where she ate with her parents before they moved to another house of their own. Despite Anna's attempts to appear always “positive,” we recorded signs of a latent depressive state characterized by uncontrollable crying, anger, and anxiety in which she felt contempt for herself and guilty. Anna felt guilt both when eating a small amount of food and while fasting. In the first case since she believed that “…if you succumb to hunger this means you are a weak person…” and in the other for the anguish she knew to throw on her parents. Anna's introspective capability become of great help for exploring her experience of guilt which was recognized as no longer linked to her current eating behavior only, but also to her disease. Getting sick with cancer was felt as betraying the trust of all her family members. Anna was obsessed by feelings of guilt towards all the family members who, because of her cancer, were all sad and worried. She was no longer able to have them “feeling well” and, because of her cancer, she did not feel as the perfect girl that brought satisfaction and pride in the whole family. She felt she had failed in her mandate.

A narration of her story with new attributions of meaning has allowed Anna to look at her whole situation under a different perspective. *“When we tell our past, in reality, we do not relive it, we rebuild it, which does not mean that we invent it. It is obvious that the difference between historical truth and narrative reconstruction lies in a strongly evocative power of memories, in the emotional dimension in which they come out to consciousness, in the presence of the other who, thanks to his active listening, becomes the witness and at the same time the conarrator of that story” *[24]. Anna had to rebuild a new world within herself, made of dreams, awareness, links, and relationships since the world she knew before could not face the impact with cancer. Such new narration of Anna's story allowed sharing her pain and struggle, enjoying the nonsense to find a new sense, and feel her vulnerability and fear. This allowed Anna to let new meanings to emerge along with the strength to live and to become active once more. There was a strong progress made in this way, that lead Anna and the therapists team through treacherous and dark streets but which also allowed the discovery of hidden treasures and breathtaking landscapes. 

The chance to meet Anna alone, to meet her parents and the whole family has encouraged the exploration of three generations allowing a reorganization of generational boundaries based on a stronger differentiation. The gradual change of the internal structure has also led to significant changes in the organization of the external family structure. Anna's parents have left their house in the suburbs and moved to a new home, closer to her mother's work and to her grandmother and aunt's houses. The families are very close but with own well-defined spaces and Anna now lives with her parents. The parental couple has found a dimension made up of two individuals and no longer of three, that is including their daughter, and Anna has been relieved of the burden of making everyone happy and regained a physiological-psychosocial functioning. Now both body and soul weights are adequate with Anna's age.

## 4. Discussion

### 4.1. Summary of Main Findings

We have described the case of a 15 year old adolescent who, albeit psychologically predisposed, displayed overt signs and symptoms of anorexia nervosa soon after beginning of a complex treatment for an advanced osteosarcoma. The associated malnutrition was so severe to hamper the time-effective delivery of chemotherapy. We reasoned whether anorexia nervosa was the most proper diagnosis for this patient or rather she could have been affected by a different type of disorder within the category of eating disorder not otherwise specified (EDNOS), a posttraumatic transient anorexia or a CACS [1, 2, 6, 7, 25, 26]. Since the patient fulfilled all the DSM-IV criteria (body weight < 85% of the expected, fear of becoming fat despite being underweight and distorted body image) except for amenorrhea, which was not evaluable, we concluded that anorexia nervosa of restrictive type was the appropriate diagnosis [1, 2]. In addition, specific food-related behaviors qualifying for EDNOS, that is, limited food choices and fear of choking or vomiting, were absent in Anna's case [1]. The alternative diagnosis of CACS was also considered. This syndrome of a multifactorial etiology is characterized by a progressive loss of skeletal muscle mass, independently from a concomitant fat mass loss, that cannot be completely reversed by conventional nutritional support [6–8]. In CACS the combination of reduced food intake and abnormal metabolism ultimately leads to progressive functional and psychosocial impairment [7]. The syndrome is often associated to signs of systemic inflammation, and its clinical progression is associated to the protocatabolic activity of a treatment-unresponsive underlying malignancy [7]. Patients with severe CACS have a low performance score and an expected survival of a few months due to the concomitant effects of malnourishment and cancer progression [6, 7]. In the case of Anna most of the diagnostic criteria for CACS were absent given that her malignancy was highly responsive to treatment which eventually cured the disease, the performance status and physical functioning were overall maintained throughout the clinical history. Moreover, malnourishment completely reverted upon resumption of appropriate eating habits and signs of hypercatabolism directly related to tumor metabolism and/or systemic inflammation were never detected.

Whether a latent preanorexic state could have been exacerbated to overt anorexia nervosa by a series of ingravescent emotional distresses experienced by Anna remains a possibility. Beyond diagnosis of cancer and initial aggressive chemotherapy, to which the patient apparently displayed a satisfactory coping attitude, overt development of anorexia symptoms in fact coincided with a “disfiguring” surgery to her right thigh. 

The case of Anna is intriguing under several aspects. First it underlines the diagnostic difficulty of defining anorexia in cancer-bearing adolescents, a population in which the DSM-IV diagnostic criteria, beyond their more recently described limitations [2], may turn not entirely applicable. For example, administration of birth control pills to protect ovaries from cytotoxic chemotherapy may hamper the proper assessment of amenorrhea [1–3]. Second, since changes in eating behaviors are common in cancer patients [5, 6], the problem only emerged, at both conscious and unconscious levels, when the family and later the patients herself recognized that malnutrition was directly compromising delivery of an adequate treatment for cancer. In such situations, where eating habits are not clearly discriminating for the diagnosis, it is essential to timely identify the typical signs of anorexia nervosa in the emotions and thoughts of the patient. Third, a complex family-based systemic approach, involving different techniques allowed the patient to complete successfully her treatment program and induced a long-term remission of the anorexic disease [17–23]. In addition, from Anna's story it appears that the systemic approach adopted was able to address core anorexia psychopathology and help the patient and her family to deal emotionally with cancer. Under this light the gloomy prognosis of the neoplastic disorder probably provided Anna with a strong motivational trigger to control her eating disorder.

### 4.2. Anorexia Nervosa and Cancer in the Adolescence

Anorexia nervosa in cancer-bearing individuals is rarely described and represents a diagnostic and therapeutic challenge [12–14]. Alterations of eating behavior are common in cancer patients and appear mainly related to the physical effects of both the disease and treatments [5, 6]. The comorbidity of anorexia nervosa with other independent medical conditions, such as neoplastic disease, associated with weight loss is unusual and published studies have been mostly focused on long-term cancer survivors, a setting in which anorexia follows cancer and does not overlap with it [27, 28]. In these cases anorexia is regarded as a post traumatic stress disorder and is usually accompanied by other symptoms including insomnia, anxiety, and depression [26, 28]. Similarly, Hoffman et al. have investigated signs of anorexia nervosa in adult cancer patients in a state of complete remission for at least 5 years, by concluding they were mostly related to a symptom framework compatible with a post traumatic psychological distress syndrome [28]. 

As a matter of fact, only four cases, including the present report, have been reported in which the diagnosis of anorexia nervosa was coincident with a diagnosis of cancer [12–14]. Main features of these patients are summarized in Table 1. In two cases diagnosis of cancer (acute lymphoblastic leukemia and osteosarcoma) preceded by about two months that of anorexia, while in another patient anorexia was diagnosed six weeks before a meningioma. In a further case, a gastrointestinal stromal tumor was diagnosed after a two-year-long period of severe food restriction and physical over exercise in a previously obese adolescent willing to adequately perform within his baseball team. In this case, the dimensions and kinetics of the tumor led the attending surgeon to speculate that cancer growth started at least one year before clinical diagnosis [14]. So in all these patients anorexia and cancer can be operationally considered as coincidental albeit medically independent. Interestingly, in all of these patients, including Anna, the malignant condition was clinically silent, either because still undiagnosed or because of a treatment-induced control, at the time of anorexia diagnosis (Table 1). A further observation is that upon appropriate treatment, anorexia was successfully managed in all of these patients. This can be partly explained by the more favorable prognosis of anorexia in younger patients and by the fact that the concomitant presence of a severe medical condition such as a malignancy might have provided a strong motivation for these patients to control their abnormal eating behavior. A similar phenomenon was observed in anorectic patients affected by nonneoplastic serious medical conditions such as HIV-related immunodeficiency [29].

The enormous distress of young patients suffering from cancer and, at the same time, from anorexia nervosa is due to the fact each illness coamplifies pains and discomforts derived from the other. It should also be reminded that adolescence itself represents the most complex stage of the life cycle [30]. In this delicate phase of growth physical changes are so significant to render the mental self-representation particularly vulnerable since the teenager has to face and simultaneously absorb physical, psychological, and social changes [30]. Adaptation to body changes is a difficult developmental task for all adolescents and may turn to a much stronger psychological impact in those suffering from cancer. Cancer and related treatments result in unexpected, rapid, uncontrollable, and unwanted body changes (swelling, skin changes, hair loss, scars, and venous catheter). Regulatory and pathological events make the integration of the self-image even more difficult. The control over the body and the hunger leads the adolescent to experience a sense of power that also affects the relationship with his/her family and the patient has the illusion to keep the self and the environment under control. In patients affected by anorexia nervosa the levels of self-esteem are strongly influenced by fitness and body weight; weight loss is seen as a breakthrough and a sign of self-discipline while the weight gain is experienced as an unacceptable loss of control [3, 4].

### 4.3. Family-Based Systemic Psychotherapy

Anorexia in Anna was managed by a two-year-long family-based psychotherapy which first unveiled the profound distortions in her family structure and, despite an early relapse, ultimately achieved a long-lasting remission of the disease [23, 31]. This is in agreement with previous finding that overall outcome of anorexia nervosa in adolescents seems to be better than that of adults [1, 3, 32, 33]. Steinhausen by comparing outcomes of anorexia nervosa in different cohorts of patients with a disease onset before and after the age of 17 years, found that recovery and chronicity rates were more favorable in younger patients who also displayed a lower mortality rate [33]. These authors attributed these more favorable outcomes to shorter symptoms duration before treatment in younger adolescents, as occurred in the case of Anna, which reduces the risk of disease chronicity, a major unfavorable factor leading to a poor outcome [33].

The DSM IV explores the patient's intrapsychic factors but, in order to reach a broader view of the problem, it is essential to widen the field of observation to the family and consider the context and, therefore, the whole system of relationships in which the patient lives. It is relevant that in most culture eating at the table is based on the observance of behavioral, emotional, and relational rules and so, for a teenager who wants to challenge his family, there is no better context to do this than during meals. Around the refusal for food there is the fight for power, rebellion, the desire to fight against an authority considered as too severe [21]. In adolescents with cancer the feeling of losing freedom is strikingly reinforced by the needs dictated by the disease and therapies. Under this light, the control over food intake may be regarded as a further reaction to the feeling of total loss of control that is now exercised by doctors and parents.

All the changes occurring during adolescence also affect the relationships with family and with the outside world. With growth the child intensifies his relationships with peers and starts to move away from parental figures, sometimes showing behaviors of great opposition and rebellion It is important to underline that all the anorexic symptoms produce, at body level, an apparent regression of the physical development, so that, for example, in a girl who is developing her secondary sexual features, this process can be stopped by the refusal of food. Consequently, the evolutionary transition from girl to woman is suspended and a temporal block of the life cycle of both the patient and the whole family may take place. 

During our psychotherapy with the patient and her family, different techniques were adopted. First, we depicted a three-generation family genogram, a graphic representation of a family tree that displays detailed data on emotional relationships among individuals. It goes beyond a traditional family pedigree chart allowing analysis of hereditary patterns and psychological factors that define relationships among family members and which may have had an influence on the patient's current state of mind [17, 18]. This allowed identifying structured distortions in the organization of patient's family and leading to a successful reorganization of both external and internal family structure. A further effort to explore messages from the inner world of Anna and her family was made through adoption of a modified version of the collage technique [19, 20]. The patient and her family were asked to search through magazines, drawings, and internet for pictures illustrating a given theme and stick them on a poster board. Through nonverbal and verbal family art tasks it was possible to define and analyze family dynamics and alliances. According to Onnis et al. work with families of anorexic and bulimic patients, the sculpture of family time (SFT) technique was utilized as a further tool for evaluation of the evolutive temporal movement of Anna's family [21].

The SFT is a nonverbal technique which allows the creation of symbolic space for all the emotions and relationships that link together the members of a family. The person identified as the sculptor places in the space the other members of the family system, choosing posture, gaze and facial expressions, in order to represent his vision of the emotional and relational family in a particular historical moment. The sculptor also places himself inside the sculpture, representing his position in the group. In this way the essence of the family experiences is condensed and projected into a three-dimensional image [31]. The peculiarity of the SFT is to ask each member of the family system to represent the family in three different time phases (the present, the future, and the past) through the creation of three different sculptures. Each member will place himself and other family members representing the way her/he sees it. The representation of the family in the present, past, and future can help to identify the presence or absence of a future dimension. Onnis et al. showed that “time suspension” does not only affect the growth of anorexics or bulimics, slowing the process of individual development, but it also involves the whole family that finds, in turn, a severe difficulties in making the evolutionary steps of its life cycle [21]. There is a strict interdependence between evolutionary processes of the adolescent and of her/his family. The first has to face a complex moment of growth and transformation while the family has to transform itself from a nest into a take-off runway [21]. 

Interestingly, the “suspension of the family time”, is also a typical event occurring cancer patients and their families [34, 35]. Soccorsi et al. underlined how cancer patients and family members try to control their own psychological evolution by blocking the family life cycle back to the stage immediately preceding the onset of cancer, in a desperate and illusory attempt to neutralize disease progression [34]. If cancer occurs in the adolescence, a moment in which the patient is being projected towards the outside world, such evolutionary phase can be suspended and the adolescent is forced to return to a previous stage characterized by the predominant need for care. Since the concept of “time” plays a key role in anorexia and cancer, the block of the temporal evolution of the family system is really impressive in situations where the teenager is a cancer patient affected by anorexia nervosa. In this case psychotherapy must also aim to reactivate the internal family time, using specific techniques such as the SFT [21]. Working with the family according to its needs could be really useful [23, 35]. In such situations it is important to provide a flexible setting that should involve meetings with all the family members and with its subsystems [35, 36]. 

Anorexia nervosa can occur in cancer-bearing adolescents and since eating behavior can be altered by disease and treatment-related physical factors, early identification of signs for such specific eating disorder in the emotions and thoughts of the patient is mandatory. Early treatment of anorexia in the context of a family-based psychotherapy may effectively manage the eating disorder [21, 23, 37–39] and help both patients and the family system to cope with the neoplastic disease. Further studies aimed at quantifying the incidence and prevalence of anorexia nervosa in adolescents with cancer are warranted.

## Figures and Tables

**Table 1:** Synopsis of reported cases of anorexia nervosa associated to cancer.

Author [ref.]	Age/sex	Tumor type	Time frame from diagnoses of cancer and anorexia	Anticancer treatment(s)	Status of cancer at the diagnosis of anorexia	Treatment strategy for anorexia	Clinical outcomes
Szajnberg et al. [12]	8/F	Acute lymphoblastic leukemia	Cancer diagnosed eight weeks before anorexia	Chemotherapy	Clinical remission	Cognitive behavior techniques	*Tumor:* isolated CNS relapse 15 months after remission *Anorexia:* resolved; no recurrence at leukemia relapse

O'Brien et al. [13]	13/F	Meningioma	Anorexia diagnosed six weeks before cancer	Surgery	Clinically silent	Psychotherapy and parental counseling	*Tumor:* 2-year continuous remission *Anorexia: *resolved; no recurrence at 2 years

Frankel and Halmi [14]	16/M	Gastrointestinal stromal tumor	Anorexia diagnosed 2 years before cancer	Surgery and imatinib mesylate therapy	Probably clinically silent at start of anorectic convictions	Cognitive behavior techniques	*Tumor:* recurrence at 3 years after resection of primary tumor; *Anorexia: *recurring pattern during cancer treatment and recurrences; resolved once tumor was in long-lasting control

De Benedetta et al. [present report]	15/F	Osteosarcoma	Cancer diagnosed nine weeks before anorexia	Chemotherapy and surgery	Clinical remission	Family-based systemic psychotherapy	*Tumor:* 4-year continuous remission *Anorexia:* resolved; no recurrence at 2 years
